# Photochemical Chain Scissions Enhance Polyethylene Glycol Biodegradability: from Probabilistic Modeling to Experimental Demonstration

**DOI:** 10.1021/acs.est.5c03567

**Published:** 2025-08-15

**Authors:** Kevin Kleemann, Madalina Jaggi, Stefano M. Bernasconi, Robert Alexander Schmitz, Andreas Künkel, Carsten Simon, Kristopher McNeill, Glauco Battagliarin, Michael Sander

**Affiliations:** † Institute of Biogeochemistry and Pollutant Dynamics, 27219 ETH Zurich, Zurich 8092, Switzerland; ‡ Geological Institute, Department of Earth Sciences, ETH Zurich, Zurich 8092, Switzerland; § 5184 BASF SE, Materials and Formulation Research, Carl-Bosch-Strasse 38, Ludwigshafen 67056, Germany; ∥ Eawag, Swiss Federal Institute of Aquatic Science & Technology, Duebendorf 8600, Switzerland

**Keywords:** polyethylene glycol (PEG), photochemical degradation, hydroxyl radicals, chain scission, molecular weight distribution, biodegradability, soil, sediment, environmental fate

## Abstract

Polyethylene glycols (PEGs), a major class of water-soluble polymers (WSPs), are widely used in diverse applications, from which PEGs may be released into the environment. This work investigates the effect of PEG reaction with photochemically produced hydroxyl radicals (^•^OH), an important environmental oxidant, on the molecular weight (MW) distribution of PEGs and their subsequent biodegradation in soil and sediment. Monte Carlo simulations demonstrated a pronounced decrease in the PEG MW after only a few ^•^OH-reaction-induced chain scissions on initial PEG molecules. The simulation results were validated by experimentally reacting ^13^C-labeled PEGs ( ${\mathrm{M̅}}_{n}$ = 6380 ± 400 Da) with photochemically produced ^•^OH to three extents and by analyzing the formed low MW PEG reaction products. Incubation of unreacted and ^•^OH-reacted PEGs in both a sediment and a soil over 150 days demonstrated increasing rates and extents of PEG biodegradation into ^13^CO_2_ with increasing ^•^OH-reaction extent and thus increasing amounts of low MW PEG products. This work underscores the importance of considering WSP MW distributions and dynamics caused by biotic or abiotic chain scission reactions when advancing a detailed understanding of WSP fate and biodegradability in natural and engineered receiving environments.

## Graphical abstract

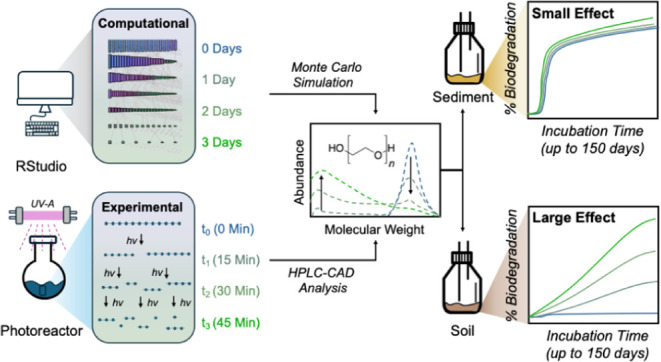

## Introduction

Polyethylene glycols (PEGs) are a major class of water-soluble polymers (WSPs) with an annual global production of approximately 600 kilotons.^1^ When used in home and personal care products as emulsifiers and moisture carriers,^2,3^ PEGs are diluted in water and are discarded into household wastewater streams. In agricultural formulations, PEGs emulsify and stabilize pesticides and fertilizers, thereby ensuring their efficient application.^4^ In both application domains, PEGs undergo dissipative release, either into wastewater treatment plants or into agricultural soils. The latter may also receive indirect inputs when fertilized with PEG-containing sludge.^5^

Despite these releases, the environmental fate of PEGs—as well as of WSPs in general—remains poorly studied and understood.^6,7^ Besides transport and mobility considerations,^8,9^ transformation processes are of particular relevance as they lead to PEG removal. Among these processes, biodegradation (i.e., PEG uptake into microbial cells and metabolic conversion into CO_2_ and microbial biomass) is the ultimate transformation reaction. Microbial metabolic utilization of PEGs is proposed to occur through exolytic (i.e., end-group specific) oxidation by three intracellular enzymes:^10–14^ an alcohol dehydrogenase, converting the terminal alcohols into aldehydes; an aldehyde dehydrogenase, oxidizing the aldehydes to carboxylic acids; and, finally, an ether bond-cleaving enzyme, releasing glyoxylate (shortening the PEG chain by one unit) which then is metabolized. Biodegradability is a highly desired trait as it would prevent PEGs from accumulating in the environment: biodegradation thus would constrain the exposure component in environmental risk assessment scenarios, irrespective of potential hazard of PEGs, which is currently assessed in academic and product safety screening studies.^15–20^

Among transformation reactions, chain scissions in PEG molecules are of great interest as they generate lower molecular weight (MW) products that, for two reasons, are expected to exhibit enhanced environmental biodegradability: first, with decreasing MW, the relative abundance of hydroxyl end groups increases, which itself is expected to increase the rate of exolytic enzymatic breakdown. Second, cellular uptake rates of PEG molecules across microbial cell walls, a key step in biodegradation, are expected to increase with decreasing PEG MW.^21^ While the effect of PEG MW on biodegradation lacks systematic study, a few published data indeed support faster biodegradation of PEGs with lower MW. For instance, PEG molecules up to 14600 Da showed complete degradation within 20 days in activated-sludge inoculated freshwater media, while PEG molecules with a number-average molecular weight of ${\mathrm{M̅}}_{n}$ = 57800 Da required 60 days of incubation for complete biodegradation.^22^ Similarly, PEGs with ${\mathrm{M̅}}_{n}$ = 400 Da showed fast biodegradation in agricultural topsoil within 42–71 days,^23^ while PEGs with ${\mathrm{M̅}}_{n}$ = 4000 Da biodegraded more slowly (estimated 735 days for 50% biodegradation to CO_2_).^24^

In sunlit aquatic systems, a major pathway that leads to PEG chain scissions and thus decreasing MW is the reaction with photochemically produced hydroxyl radicals (^•^OH). The rate of this reaction increases with increasing steady-state ^•^OH concentrations ([^•^OH]­ss) over the reported range from 10^–17^ M to 10^–15^ M.^25–30^ Major environmental photosensitizers for ^•^OH are nitrate, nitrite, and dissolved organic matter (DOM).^26,31,32^ PEG reaction with ^•^OH is well-studied for water treatment in which ^•^OH is generated from UV/H_2_O_2_ treatment and ozonation. These past studies have shown that PEG chain scissions lower ${\mathrm{M̅}}_{n}$ while increasing the dispersity (*D̵*_M_ = weight-average molecular weight ${\mathrm{M̅}}_{w}$ over ${\mathrm{M̅}}_{n}$ ) of PEGs.^33,34^ By contrast, PEG reaction with ^•^OH at environmentally relevant [^•^OH]­ss and the resulting hypothesized increased biodegradability remain unexplored.

This work systematically assessed the effects of ^•^OH-reaction induced chain scissions on the MW distribution and subsequent biodegradability of PEGs in soil and sediment. PEG chain scissions were hypothesized to enhance biodegradability. This work followed a three-step approach: First, numerical Monte Carlo simulations were run to predict ^•^OH reaction-induced decreases in the average MW of MW-distributed PEG molecules. Second, Monte Carlo simulations were verified experimentally by reacting ^13^C-labeled PEGs $({\mathrm{M̅}}_{n}=6380\pm 400\;\;Da$ ) with photogenerated ^•^OH to three reaction extents, combined with analyzing resulting low-MW PEG molecules using high-performance liquid chromatography coupled to both a charged aerosol detector and a mass spectrometer (HPLC-CAD-MS). Third, the initial unreacted PEG solution as well as the three PEG solutions with increasing ^•^OH reaction extents were incubated in a soil and a sediment. Biodegradation was followed by quantifying both PEG mineralization to ^13^CO_2_ and the residual PEG-added ^13^C in soil and sediment after the incubations. This study links PEG reaction-induced chain scissions to its environmental biodegradability. This work is the first to use stable carbon isotope labeling for WSP biodegradability assessment, extending on previous work using this approach for biodegradable structural polymers.^35–38^

## Materials and Methods

### Software Declaration

All simulations, data analysis, and plotting were conducted using R (version 4.4.0) via RStudio (version 2024.0X).

### Reaction Kinetics of PEG with ^•^OH

PEG reaction with ^•^OH was first modeled assuming pseudo-first-order reaction kinetics (eq 1) at three [^•^OH]_SS_ of 10^–15^ M, 10^–16^ M, and 10^–17^ M, which fall into the concentration range of sunlit surface waters.^26,28^

k1=kM·[OH•]SS 1

where *k*_1_ (s^–1^) is the pseudo-first-order reaction rate constant, and *k*_M_ (=2.1 × 10^9^ M^–1^ s^–1^) is the experimental rate constant for ^•^OH with a monomeric unit in dissolved PEG.^39^

Rate constants for reactions following ^•^OH attack that lead to chain scission are higher than *k*_1_.^33^ The overall rate of chain scission, therefore, is close to being diffusion-limited (since *k*_diff_ is usually around 10^–9^–10^–10^ M^–1^ s^–1^ according to the Smoluchowski equation).

We parametrized the extent of PEG backbone cleavages by defining an average number of scissions per initial PEG chain—abbreviated in form of the dimensionless variable $\overset{®}{SiC}\left(t\right)$ —as the fraction of reacted monomeric units divided by the average degree of polymerization (e.g., average number of monomeric units in the chains). Under the assumption that all monomeric units for a given PEG chain have the same probability of reacting with ^•^OH, $\overset{®}{SiC}\left(t\right)$ is given by eq 2.

$$\overset{®}{SiC}\left(t\right)=\frac{\left(1-e^{-k_{1}\cdot t}\right)\cdot {\mathrm{M̅}}_{n}\left(t_{0}\right)}{{MW}_{Monomer}}$$ 2

where *t* (s) is the reaction time, ${\mathrm{M̅}}_{n}$ ­(*t*_0_) is the initial number-average MW of the PEG molecules, and MW_Monomer_ is the MW of the PEG monomeric unit (i.e., 44 Da). The $\overset{®}{SiC}\left(t\right)$ were converted into time-dependent, number-averaged molecular weights, ${\mathrm{M̅}}_{n}$ (*t*), using eq 3 (validated previously to describe polymer chain scissions^34,40,41^ )­

3 $${\mathrm{M̅}}_{n}\left(t\right)=\frac{{\mathrm{M̅}}_{n}\left(t_{0}\right)}{\overset{®}{SiC}\left(t\right)+1}$$

### Probabilistic Monte Carlo Simulation of PEG Chain Scissions

An ensemble of *m* total PEG chains with defined lengths of *n* monomeric units was defined. The fraction of total PEG chains with length *n*, denoted as *p*(*n*), followed a normal distribution centered around a characteristic mean chain length, λ, with a spread determined by the standard deviation σ. Therefore, the number of individual PEG chains with length *n*, *m*(*n*), was given as *m*(*n*) = *p*(*n*) · *m*. Subsequently, a stochastic chain scission was simulated. To this end, an individual PEG chain *i* (*i* ∈ {1, 2, 3, ..., *m*}) was selected for cleavage with a selection probability *P*_i_ that matched its chain length-specific reaction rate constant, *k*(*n*_i_) (M^–1^ s^–1^), as described in eq 4

4 $$P_{i}=\frac{k\left(n_{i}\right)}{\sum_{j=1}^{m}k\left(n_{j}\right)}\;where\;k\left(n\right)=\{\begin{array}{l} 0.8\cdot k_{M}\cdot n\;for\;n\leq 30\\ 0.8\cdot k_{M}\cdot 30\cdot {\left(\frac{n}{30}\right)}^{0.57}for\;n>30 \end{array}$$

where *k*(*n*) is derived as detailed in Section S1 in the Supporting Information, and *k*_M_ (M^–1^ s^–1^) is the intrinsic reaction rate constant of a PEG monomeric unit with ^•^OH. For the selected PEG chain *i*, a reaction site along the backbone chain was randomly ascribed. The reaction was simulated to result in chain scission into two shorter PEG chains, with their combined lengths equaling the length of the PEG chain undergoing scission. The two formed PEG chains then replaced the reacted PEG chain for the next simulation step (resulting in an increase in the total number of chains of *m* + 1 per scission event). Overall, the total chain number and chain length distribution was dynamically updated during the multistep MC simulation. Ethylene glycol (monomeric unit) was the final end product of the simulated chain scission process.

### Photodegradation Experiments

Triplicate solutions of ^13^C-PEG (Polymer Source Inc., ${\mathrm{M̅}}_{n}$ = 6380 ± 400 Da) were prepared for each reaction time point (*t*_0_ = 0 min (unreacted solution), *t*_1_ = 15 min, *t*_2_ = 30 min, and *t*_3_ = 45 min) by adding 6 mL of a ^13^C-PEG solution (5 mg ^13^C-PEG mL^–1^; prepared in Milli-Q (MQ) water; resistivity >18 mΩ cm; Barnstead NANOpure) and 2 mL of a H_2_O_2_-solution (100 mM H_2_O_2_ in MQ) into glass tubes (final concentrations of 3.75 mg mL^–1 13^C-PEG and 25 mM H_2_O_2_). The tubes were placed on a carousel inside a photoreactor (LZC-4 V, Luzchem, Canada) equipped with twelve 365 nm UVA broad band bulbs and irradiated for the above-specified time. Dark (nonirradiated) controls with the same initial PEG and H_2_O_2_ concentrations were run to validate that PEGs did not degrade in the absence of photochemically produced ^•^OH. Following removal of triplicate tubes from the reactor, the solution volume in each tube was split into three subsamples: 100 μL for HPLC-CAD-HRMS analysis, 900 μL for ^13^C NMR analysis, and 7 mL for the biodegradability testing. The HPLC-CAD-HRMS method and setup as well as the ^13^C NMR analysis are described in detail in Section S2, Supporting Information. For HPLC-analyzed solutions with PEG concentrations <1 mg mL^–1^, the response of the CAD is proportional to total PEG mass and independent of PEG MW,^42^ allowing for quantification of PEG molecules over a wide range of *n*. Details on the biodegradability testing are provided below.

### Sediment and Soil Incubations

#### Preparations

Residual H_2_O_2_ in the 7 mL subsamples of photoirradiated ^13^C-PEG solutions (*t*_0_ to *t*_3_) was removed by adding 0.05 mL of catalase solution (Bovine liver catalase; Sigma-Aldrich, 0.44 mg protein mL^–1^; activity of 1 × 10^4^ to 4 × 10^4^ U mg^–1^ protein). Subsequently, the unreacted and reacted ^13^C-PEG solutions were added into the soil and sediment incubation bottles (see below). Control solutions without ^13^C-PEG but the same H_2_O_2_ starting concentration were treated analogously with catalase before being added to blank incubation bottles. Triplicate bottles were prepared for each incubation medium (i.e., *t*_0_ to *t*_3_ and ^13^C-PEG-free blank), resulting in a total of 15 bottles for each sediment and soil incubations.

For sediment incubations, a sediment core was collected in Lake Rotsee (Lucerne, Switzerland; sampling date: 27^th^ September 2023; GPS coordinates: 47.07°N, 8.32°E; water column depth of 16 m). The top 5 cm of this core was mixed with an equal volume of lake water to a total volume of approximately 2 L and stirred for 1 h. Under continuous stirring of the sediment suspension, 40 mL aliquots (approximately equaling 3 g sediment dry weight) were transferred into 100 mL Schott incubation bottles. All bottles were placed in an incubation chamber at 20 °C and continuously stirred over the course of the incubation.

Standard soil 6S (Landwirtschaftliche Untersuchungs- and Forschungsanstalt (LUFA), Speyer, Germany; collected in October 2023) was sieved to 2 mm. Physicochemical properties of soil S6 are provided in Section S3, Supporting Information. The soil water content was adjusted to 50% of its maximum water holding capacity (=41.4 wt %) using MQ water. The soil added to each incubation bottle had an equivalent dry weight of 20 g.

The prepared sediment and soil were preincubated for 7 days prior to dropwise addition of the 7 mL of either the respective ^13^C-PEG solutions (*t*_0_ to *t*_3_) or the ^13^C-PEG-free blank solution. The incubations were run for a total of 150 days at 20 °C. Following the first 4 weeks of incubation, water contents in the soil and sediment incubation bottles were checked biweekly by weighing of the bottles. Water loss through evaporation was compensated by adding appropriate amounts of MQ water to the bottles.

#### Quantification of PEG-Derived ^13^CO_2_ during Incubations

Mineralization of ^13^C-PEGs to ^13^CO_2_ was followed using an automated analysis system as previously described.^36^ Slight modifications of this incubation setup are described in Section S4, Supporting Information.

#### Closing PEG Carbon-13 Mass Balances for Incubations

After terminating the incubations, the nonmineralized residual PEG-added ^13^C, ^13^C_Non‑Mineralized_ (which may include both ^13^C incorporated into microbial biomass and ^13^C in residual PEG), was quantified for each incubation bottle. To this end, the sediment or soil from each bottle was freeze-dried and subsequently milled (30 Hz, 1 min, MM400 oscillatory ball mill, Retsch GmbH & Co). A 10 mg aliquot from each milled sediment and soil was taken for elemental analysis (EA) coupled to isotope ratio mass spectrometry (IRMS). ^13^C_Non‑Mineralized_ was quantified as described previously^36^ and as detailed in Section S5, Supporting Information.

## Results and Discussion

### Modeling MW Decreases of PEGs upon Reaction with ^•^OH

#### Pseudo-First-Order Reaction Kinetic Modeling

In a first approach, we used pseudo-first-order reaction kinetics (eq 1) to model the rates of ^•^OH-induced chain scissions in three theoretical PEG mixtures with initial number-average molecular weights of M̅ _n_ = 12, 6, and 3 kDa. As expected, the scission rate of PEGs in these mixtures increased with increasing [^•^OH]_SS_ (Figure 1a,b). For high environmental [^•^OH]_SS_ of 10^–15^ M (Figure 1a—light colors), the average number of scissions per initial PEG chain, $\overset{®}{SiC}$ , ranged from 4 (initial M̅ _n_ = 12 kDa) to 20 (initial M̅ _n_ = 3 kDa) after 1 day of reaction. By comparison, for low environmental [^•^OH]_SS_ of 10^–17^ M (Figure 1a—dark colors), 10 days of reaction resulted in $\overset{®}{SiC}$ of only 0.5 to 2 for initial M̅ _n_ of 12 and 3 kDa, respectively. The M̅ _n_ decreased exponentially from the initial 12, 6, and 3 kDa to M̅ _n_ < 1 kDa within a few days for [^•^OH]_SS_ = 10^–15^ M and to approximately M̅ _n_ = 5, 3.5, and 2.3 kDa within 10 days for [^•^OH]_SS_ = 10^–17^ M. These results show that ^•^OH-induced reactions can significantly lower the MW of PEGs in sunlit surface waters over residence times of hours to a few days. We used the calculated environmentally realistic $\overset{®}{SiC}$ values from 1 to 8 as termination criteria in the subsequent detailed simulations.

**Figure 1:**
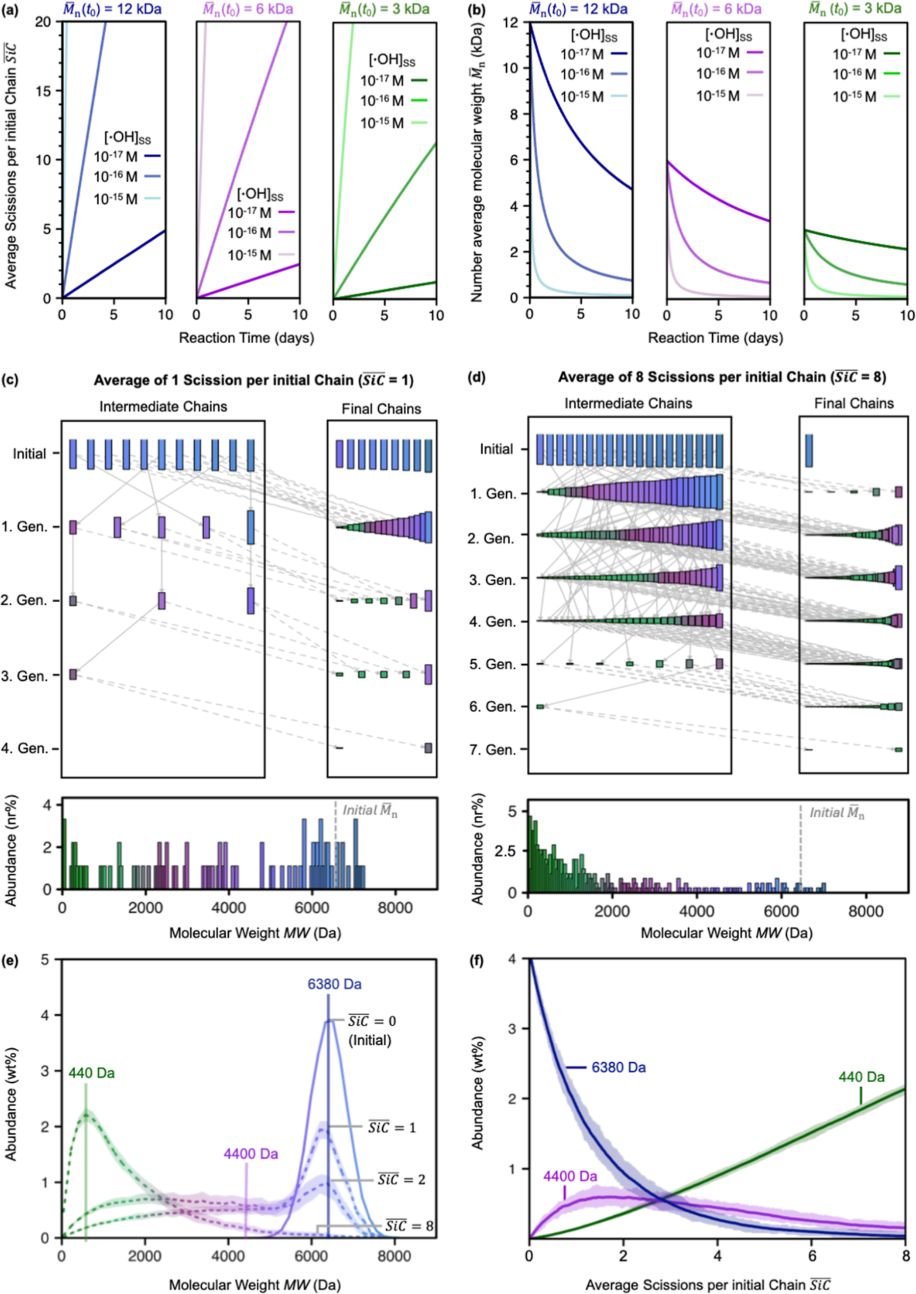
(a,b) Pseudo-first-order kinetic modeling of the reaction of polyethylene glycol (PEG) with hydroxyl radicals (^•^OH) results in linear increases in the average number of scissions per initial chain ( $\overset{®}{SiC})$ within a reaction time of 10 days (panel a), and an exponential decay of the number-average molecular weight ( ${\mathrm{M̅}}_{n}$ ) with reaction time *t* (panel b), calculated for three initial PEG ${\mathrm{M̅}}_{n}\left(t_{0}\right)$ of 12 kDa (blue traces), 6 kDa (purple traces), and 3 kDa (green traces) at three environmental ^•^OH steady-state concentrations of [^•^OH]_SS_ = 10^–15^ M, 10^–16^ M, and 10^–17^ M (shades of the corresponding color). (c,d) A simulated reaction network plot illustrating the results of single Monte Carlo simulations with 20 initial PEG chains and simulation termination criteria of $\overset{®}{SiC}$ = 1 and 8 in panels c and d, respectively, with the resulting nr % (i.e., the number of PEG molecules with a given molecular weight (MW) in percent of the total number of chains obtained) contributions of individual PEG molecules of given MW plotted below and the initial MW distribution shown as a dashed line. The PEGs are color-coded according to MW ranging from high (blue) to medium (purple) to low (green) MWs. (e,f) Averaged results of 50 Monte Carlo simulations on 5000 initial PEG molecules with an initial MW distribution of 6380 ± 400 Da. Simulation results are depicted as weight percent (wt %) distributions of the MWs of PEG molecules after terminating simulations at $\overset{®}{SiC}$ of 1, 2, and 8 (relative to the initial distribution; $\overset{®}{SiC}$ = 0). Panel f shows the changes in the wt % contribution of PEG molecules with selected MWs (i.e., MW = 6380 Da (blue), MW = 4400 Da (purple), and MW = 440 Da (green)) with increasing simulated $\overset{®}{SiC}$ . The 95% confidence intervals of the simulated outputs are shown as shaded areas around the averages in panels (e,f).

#### Monte Carlo Simulations

Figure 1c,d illustrate the outcomes of single simulations of ^•^OH-induced chain scissions on an initial ensemble of 20 PEG chains up to a $\overset{®}{SiC}$ of 1 and 8 (i.e., corresponding to a total of 20 and 160 chain scissions, respectively). The lengths of the initial 20 chains were set to match the MW distribution of the ^13^C-labeled PEG mixture (i.e., MW = 6380 ± 400 Da, average ± standard deviation) used in the experimental part below. For the shown simulation terminated at $\overset{®}{SiC}$ = 1, only 11 of the initial 20 PEG molecules had reacted with ^•^OH (Figure 1c). These 11 molecules were thus labeled as “Intermediate Chains”, whereas the 9 initial molecules that did not react over the simulation were classified as “Final Chains” (i.e., they made up part of the final ensemble of PEG molecules at the end of the simulation). When reacting, each of the intermediate chains formed two lower-MW daughter products, as depicted by two arrows pointing away from each “intermediate chain”. Daughter products originating from an initial chain are denoted as first generation intermediates. Note that the numbering of generation intermediates does not reflect the chronological order of their formation (e.g., a first generation intermediate from an initial PEG molecule can form late in the simulation) but instead the total number of consecutive chain scissions needed for their formation. For the given simulation, the reacted 11 PEG molecule formed five “Intermediate Chains” that subsequently continued to react with ^•^OH. The remaining 17 daughter products (i.e., 2 · 11–5) were part of the “Final Chains” pool as they were not selected to react further in this simulation. A total of 9 “Intermediate Chains” formed up to the third generation (Figure 1c) when the simulation was terminated at $\overset{®}{SiC}$ = 1. The simulated MW distribution of the PEG molecules in the “Final Chains” pool is shown below the network plot in nr % abundance (i.e., the number of PEG molecules with a given MW over the total number of chains obtained) versus MW.

An analogous simulation with $\overset{®}{SiC}$ = 8 as the termination criterion resulted in the reaction of 19 out of the 20 initial PEG molecules and the formation of daughter products up to the seventh generation (Figure 1d). Most “Final Chains” belonged to the second to fourth scission generations. The most abundant formed PEG molecules had MWs below 2000 Da.

For reliable statistics and better-resolved MW distributions of the final ensemble of PEG molecules, a total of 50 simulations with 5000 initial PEG molecules (maintaining the MW distribution of 6380 ± 400 Da) were run per $\overset{®}{SiC}$ termination scenario and averaged. Simulations terminated at $\overset{®}{SiC}$ = 1 showed that a large number of the initial PEG molecules did not react, as evidenced from the high abundance and the remaining bell-shaped distribution around the starting average MW of 6380 Da (Figure 1e; note that the final MW distribution of PEG molecules is now expressed in weight percent (wt %) to capture the actual mass distribution). At the same time, a substantial fraction of formed PEG molecules had MWs between 1000 and 5000 Da. Simulations up to at $\overset{®}{SiC}$ = 2 further lowered the abundance of unreacted PEG molecules and increased the abundance of formed PEG molecules with MW between 1000 and 5000 Da. Finally, simulations terminated at $\overset{®}{SiC}$ = 8 resulted in extensive depletion of initial PEG molecules and a final ensemble of PEG molecules with MWs predominantly below 4000 Da.

Figure 1e,f show that there were three principal PEG reaction domains across the selected MW range. First, the abundance of high-MW PEG molecules (highlighted for the exemplary PEG with MW = 6380 Da; blue lines) continuously decreased with increasing $\overset{®}{SiC}$ s, reflecting a higher probability of loss through reaction than formation through chain scissions on even higher-MW PEG molecules. Second, PEG molecules with intermediate MWs (highlighted for an exemplary PEG with MW = 4400 Da; purple lines) exhibited an increase in relative abundance up to $\overset{®}{SiC}$ = 1.5, reflecting a lower probability of reacting than formation. Beyond $\overset{®}{SiC}$ = 1.5 and the continuous depletion of higher MW PEGs, the probability of the intermediate-MW PEGs to react exceeded that of being formed, resulting in a slow and continuous decrease in their relative abundance with increasing $\overset{®}{SiC}$ . Third, the abundance of low-MW PEG molecules (e.g., PEG with MW = 440 Da; green lines) continuously increased with increasing $\overset{®}{SiC}$ , reflecting the higher probability of these PEG molecules forming than reacting.

Compared to the pseudo-first-order rate modeling of decreases in PEG ${\mathrm{M̅}}_{n}$ (Figure 1a,b), the Monte Carlo simulations clearly offer the advantage of resolving MW changes in individual PEG molecules over the course of the reaction. For a given $\overset{®}{SiC}$ as termination criteria, the individual PEG molecules can be combined into the final ensemble (Figure 1c–f). The simulation provides information on the MW of all individual PEG molecules and their relative abundance, which is critical for assessing the hypothesized MW-dependence of PEG biodegradation.

### Experimental Analysis of MW Decreases of PEGs upon Reaction with ^•^OH

A ^13^C-PEG solution ( ${\mathrm{M̅}}_{n}$ = 6380 ± 400 Da) was reacted with ^•^OH from H_2_O_2_ photolysis for *t*_1_ = 15 min, *t*_2_ = 30 min, and *t*_3_ = 45 min. While ^•^OH is the dominant reactive intermediate formed in the UV-A/H_2_O_2_/PEG system explored in this work, superoxide may also have formed via decomposition of PEG-hydroperoxide intermediates. However, given that superoxide does not react with saturated ethers, we do not expect it to have contributed to PEG photodegradation. The choice of ^13^C-PEGs instead of nonlabeled PEGs was motivated by subsequent biodegradability assessments (see below). An exemplary HPLC-CAD chromatogram of PEG molecules in the *t*_1_ solution is shown in Figure 2a. The entire set of chromatograms including the initial PEG solution (*t*_0_ = 0 min; no photolysis) and the three reaction times (*t*_1_ to *t*_3_) are provided in Section S2, Supporting Information. The chromatograms show that individual PEG molecules were well separated over a large MW range from 2000 to 6400 Da, using parallel HRMS analysis to allow for peak assignment to specific PEG molecules of defined MWs (Figure 2a). For illustration, Figure 2b shows the elugrams for three targeted ions (i.e., mass (*m*) to charge (*z*) ratios of *m*/*z* = 1758, 1873, 1988; all with *z* = 2) in the HRMS that correspond to PEG molecules with *n* = 76, 81, and 86 repeat units (calculated using an MW = 46.05 Da of the ^13^C_2_-repeat unit). The exact PEG masses were assigned based on the corresponding HRMS spectra (Figure 2c). Separation of individual PEG molecules was compromised at higher mass loadings onto the HPLC column (e.g., PEGs with MW > 5000 Da and MW < 3000 Da for the *t*_1_ solutions). As a result, integral calculations over these MW regions showed higher uncertainties (as further discussed below). Separation using the current analytical setup was insufficient for PEGs with MWs below approximately 2000 Da and above approximately 6400 Da (filled green and purple peak areas in Figure 2a, respectively).

**Figure 2:**
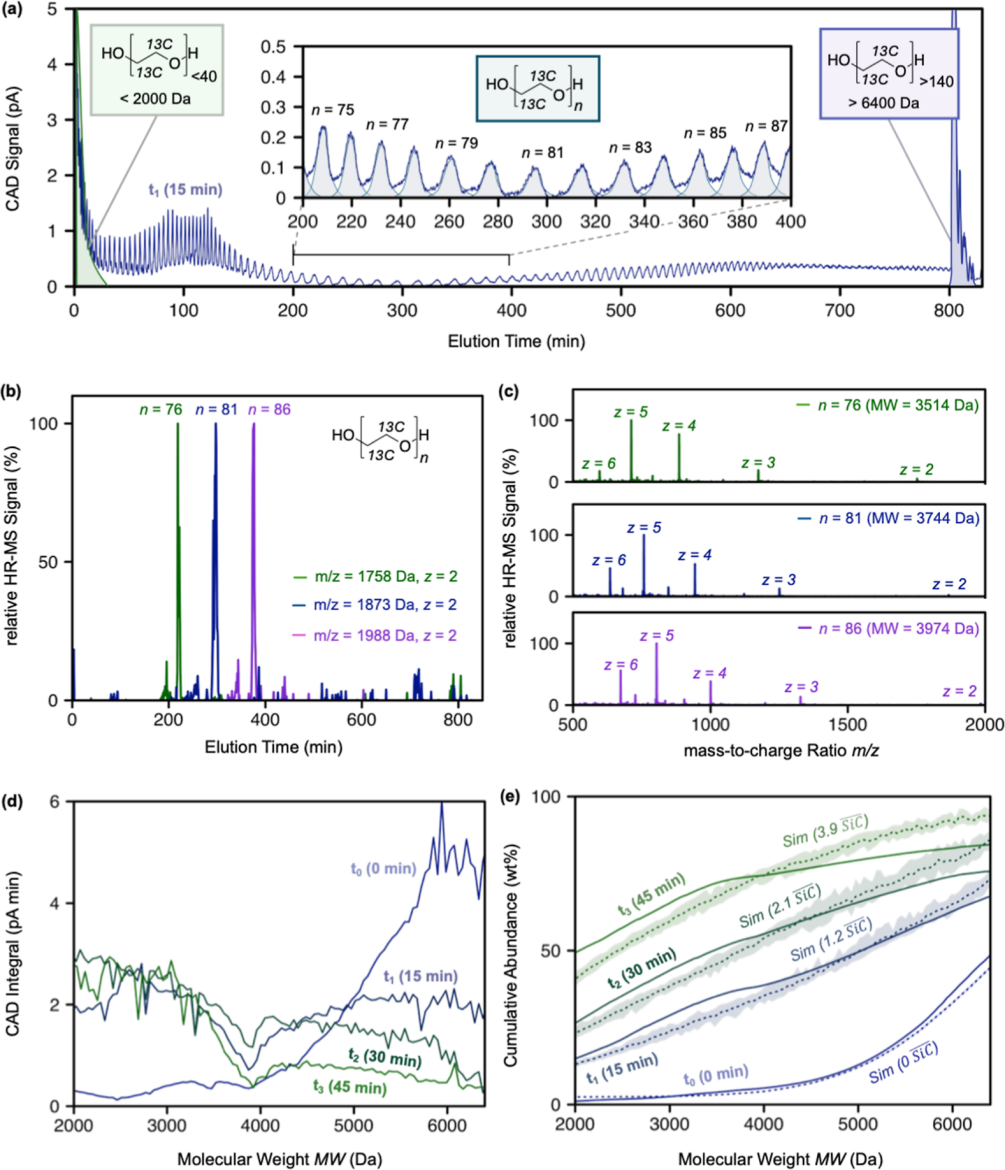
(a) Chromatogram of the ^13^C-polyethylene glycol (PEG)-containing solution after a reaction time of *t*_1_ = 15 min with hydroxyl radicals (^•^OH), as analyzed by high-pressure liquid chromatography coupled to charged aerosol detection (HPLC-CAD). The retention time window from 200 to 400 min is shown in the inset, highlighting the well-separated peaks for individual ^13^C-PEG molecules with repeat unit numbers, *n*, from *n* = 75 (corresponding to a molecular weight (MW) of 3468 Da) to *n* = 87 (MW = 4020 Da). Peak integrals are given in light blue in the inset. PEG molecules with MWs below 2000 Da (green peak) and above 6400 Da (purple peak) are highlighted. The peaks detected by CAD were assigned to PEG molecules with specific MWs by parallel high-resolution mass spectrometry (HRMS) detection (see panels b,c and all spectra in Section S2, Supporting Information). (b) HRMS signal chromatogram of three individual ion traces with mass-to-charge ratios of *m*/*z* = 1758 (green trace), 1873 (blue trace), and 1988 (purple trace), with intensities normalized to the highest peaks. These peaks correspond to the elution of PEG molecules with *n* = 76, 81, and 86 repeat units (all *z* = 2). (c) Mass spectra of the eluting peaks with highest signal intensity in panel (b), showing the mass of the eluted PEG at different molecular charges *z*. (d) CAD peak integrals for each identified PEG over the MW range from 2000 to 6400 Da for the unreacted solution (i.e., *t*_0_ = 0 min) and for the solutions reacted for *t*_1_, *t*_2_, and *t*_3_ of 15, 30, and 45 min, respectively. (e) Cumulative abundance of PEG molecules as a function of MW, with abundance being calculated as the cumulative sum of CAD peak integrals up to the given MW normalized to the summed areas of all CAD peaks over the entire MW range (solid lines), including the poorly resolved features at <2000 Da and >6400 Da. The dashed lines and corresponding shades represent the best fits of the Monte Carlo simulation and their 95% confidence intervals to the experimental data.

Integration of the CAD peaks in the *t*_0_-sample chromatogram revealed that approximately 50 wt % of the ^13^C-PEG molecules were below the ${\mathrm{M̅}}_{n}$ = 6380 Da as specified by the supplier (Polymer Source Inc., Dorval, QC, Canada), indicating a symmetric MW distribution (*t*_0_ lines in Figure 2d,e). The smallest ^13^C-PEG molecules in the t_0_ samples had MWs in the range of 4000 Da (Figure 2d,e). As expected, reaction with ^•^OH led to the formation of smaller PEGs: the abundance of PEG molecules with MW < 2000 Da (*y*-axis intercepts in Figure 2e; obtained by integration of the poorly resolved early peak in the sample chromatogram (Figure 2a)) increased from 0 wt % in the *t*_0_ sample to 18, 25, and 49 wt % at *t*_1_, *t*_2_, and *t*_3_ samples, respectively. At the same time, the abundance of the high-MW fraction >6400 Da decreased from approximately 50 wt % in *t*_0_ to 30, 25, and 20 wt % in the *t*_1_, *t*_2_, and *t*_3_ solutions, respectively. The abundance of PEGs with MW > 6400 Da, however, may have been slightly overestimated as the MS spectra revealed that also a few small PEG molecules eluted in the high-MW elution time region of the chromatogram (labeled >6400 Da in Figure 2a). At the same time, the summed integrals of all peaks in the sample chromatograms, including the poorly resolved peaks for MW < 2000 Da and MW > 6400 Da (Figure 2a), yielded similar values for all four tested solutions, indicating that reaction of PEG with ^•^OH resulted in little (if any) photomineralization in the current experiment. Overall, with increasing reaction time from *t*_1_ to *t*_3_, the MW distribution of PEG molecules became more uniform across the MW range from 2000 to 6400 Da (Figure 2d).

Fitting the experimentally determined cumulative MW distribution data (solid lines) by results of Monte Carlo simulations run in increments of 0.1 $\overset{®}{SiC}$ (parameters as specified above, dashed lines; partial least-squares residual fitting) resulted in best fits with estimated $\overset{®}{SiC}$ of 1.2, 2.1, and 3.9 for *t*_1_, *t*_2_, and *t*_3_, respectively (Figure 2d). The overall good agreement between the fits and the experimental data suggests that the kinetic and stochastic nature of PEG chain scissions by ^•^OH were accurately captured in the simulations. This agreement highlights that chain scission extents obtained in the photoreactors can be achieved at environmentally realistic [^•^OH]_SS_ over reasonable time frames. The slightly higher fitted than experimentally measured abundance of PEG scission products with MW > 4500 Da likely reflected that the concentrations of PEGs in this MW range (particularly in the *t*_3_ solution) were low and close to the limit of quantification of the HPLC-CAD method. More importantly, the fitting results revealed that the experimental design resulted in realistic environmental scenarios (i.e., $\overset{®}{SiC}$ from 1 to 4), in good agreement with $\overset{®}{SiC}$ values resulting from [^•^OH]_SS_ = 10^–16^ M and residence times between 12 h and 3 days (Figure 1a).

HRMS spectra of *t*_1_ to *t*_3_ solutions contained peaks with a positive mass offset of 16 Da relative to the original ^13^C-PEG molecular masses, indicating some reaction products in which a hydrogen in the PEG was replaced by a hydroxyl group. Furthermore, the intensities of peaks with a −2 Da mass difference increased with increasing reaction time, suggesting end-group aldehyde formation. These transformations were confirmed by ^13^C NMR spectra collected from aliquots of the initial and the three irradiated PEG solutions (Section S2, Supporting Information, for spectra). In addition, the ^13^C NMR spectra suggested the formation of small amounts of in-chain ester bonds and low-MW products. Thus, the HRMS and ^13^C NMR data align with the mechanisms and product formation previously proposed for PEG reaction with ^•^OH in engineered systems.^33,34,43^ While spectral overlap and small absorbance peaks impaired quantification of oxidation products, their small peak areas suggested that they were formed in only small amounts—and thus likely only had minor contributions to mineralization data in subsequent biodegradability tests (see below).

### Effect of PEG MW Reduction via ^•^OH Reaction on PEG Biodegradability

We tested the hypothesis of enhanced biodegradability of lower-MW PEG scission products by incubating aliquots of the unreacted PEG solution (i.e., *t*_0_ = 0 min) and the three reacted PEG solutions (i.e., *t*_1_, *t*_2_, and *t*_3_ = 15, 30, and 45 min, respectively) in Lake Rotsee sediment and LUFA 6S reference soil. The use of ^13^C-labeled PEGs enabled the selective quantification of PEG mineralization to ^13^CO_2_ (i.e., ^13^C_Mineralized_) over the course of the incubations, as well as the total nonmineralized PEG-added ^13^C (i.e., ^13^C_Non‑Mineralized_) remaining in the sediment and soil at the end of the incubations.

#### Sediment Incubations

Addition of the unreacted ^13^C-PEG *t*_0_-solution to the sediment resulted in low initial mineralization rates which increased to a maximum rate of 0.97 ± 0.32% ^13^C h^–1^ at around 6 days of incubation, after which rates continuously decreased (Figure 3a; rates shown only for the first 20 days of incubation). Time-integration of the mineralization rates over the entire incubation period of 150 days resulted in final cumulative mineralization extent of ^13^C_Mineralized_ (*t*_0_ sample) = 66 ± 3% (Figure 3b). The incubations of the three reacted *t*_1_ to *t*_3_ solutions also showed maximum mineralization rates of comparable values at around 6 days of incubation. However, each of the three reacted PEG solutions showed an additional maximum in ^13^C-PEG-mineralization rates at 2 days of incubation. For these peaks, the maximum rates increased from 0.1% ^13^C h^–1^ for *t*_1_ to approximately 0.3% ^13^C h^–1^ for *t*_3_. We ascribe these new, additional peaks to the microbial utilization of low-MW PEG molecules formed through reaction with ^•^OH. The cumulative mineralization extents after 3 days of incubation also increased noticeably from ^13^C_Mineralized_ = 4 to 7 and 9% from *t*_1_, to *t*_2_, and *t*_3_, respectively. This increase in mineralization extent with reaction time was maintained throughout the entire incubation with final ^13^C_Mineralized_ increasing from 71 ± 0.5% for *t*_1_, to 76 ± 4.1% for *t*_2_, and 79 ± 3% for *t*_3_ samples, all higher than the ^13^C_Mineralized_ = 66 ± 3% for the *t*_0_ sample (Figure 3b).

**Figure 3:**
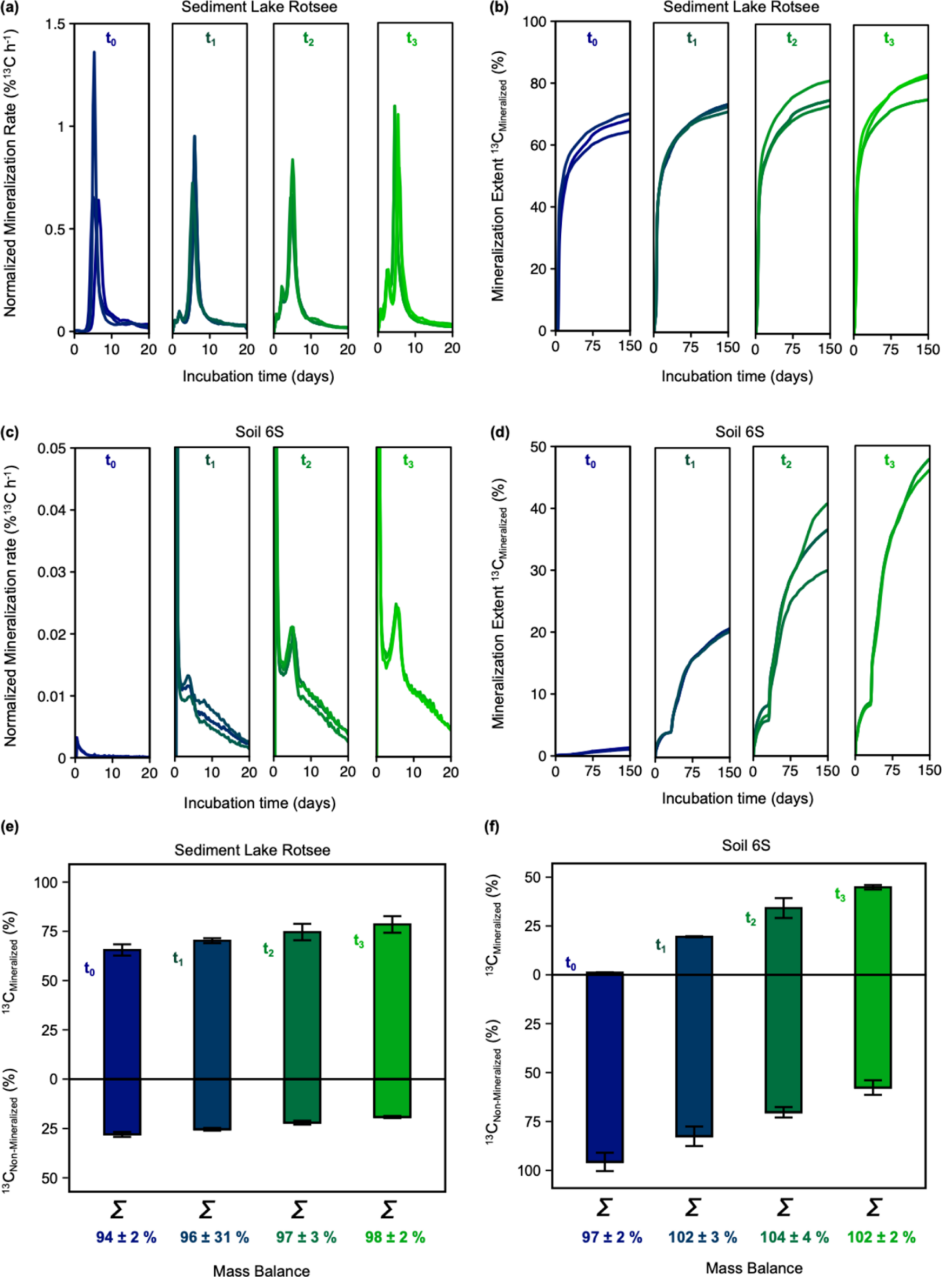
Sediment and soil incubations to determine biodegradation of ^13^C-labeled polyethylene glycol (PEG) molecules that either were unreacted (i.e., *t*_0_ = 0 min; dark blue; with a molecular weight (MW) distribution of 6380 ± 400 Da) or reacted with ^•^OH for increasing durations of *t*_1_ = 15 min (dark cyan), *t*_2_ = 30 min (dark green), and *t*_3_ = 45 min (light green) and thus containing increasing amounts of low-MW PEG molecules. (a,c) Mineralization rates of ^13^C-PEG to ^13^CO_2_ normalized to the total added PEG-^13^C (expressed in % of added ^13^C per hour) and (b,d) the corresponding cumulative mineralization extents, ^13^C_Mineralized_, expressed as CO_2_–^13^C formed in percent of initially added PEG-^13^C. Incubations in sediment of lake Rotsee (water-dispersed slurries, continuously stirred) and soil LUFA 6S (unsaturated pore water conditions, static) were run at 20 °C under oxic conditions. Nonmineralized PEG-added ^13^C that remained in the sediment of lake Rotsee (e) and soil LUFA 6S (f) at the end of the incubations (i.e., ^13^C_Non‑Mineralized_, in ^13^C in percent of total added PEG-^13^C) plotted with the corresponding cumulative mineralization extents at the end of the incubations (i.e., ^13^C_Mineralized,_ as ^13^CO_2_ in percent of total added PEG-^13^C; identical to final ^13^C_Mineralized_ data shown in panels (b,d). Mass balances on PEG-added ^13^C (i.e., ^13^C_Mineralized_ + ^13^C_Non‑Mineralized_ in % of total added PEG-^13^C) were closed as shown by numbers close to 100% below the panels. Incubations were generally run in triplicate, except for *t*_0_ (dark blue, LUFA 6S soil) which was run in duplicate due to limited incubator space. Although *t*_3_ (light green, LUFA 6S soil) was initially run in triplicate, one incubation line malfunctioned, so only duplicate data were reported. Individual triplicate data are shown in panels (a–d), while averages and standard deviations (error bars) are shown in panel e and panel f.

After termination of the incubations, EA-IRMS analysis of sediment aliquots from all bottles yielded decreasing ^13^C_Non‑Mineralized_ from *t*_0_ to *t*_3_ samples (Figure 3e). More importantly, the summed ^13^C_Non‑Mineralized_ and ^13^C_Mineralized_ corresponded to between 96 ± 1% and 98 ± 2% of the total PEG-^13^ C initially added into the incubation flasks, demonstrating closed mass balances on PEG-added ^13^C (Figure 3e). The ability to verify closed polymer carbon mass balances by allowing to delineating polymer-derived ^13^C from carbon in sediment (and soil) organic matter highlights the power of using ^13^C-labeled polymers in biodegradation studies, offering a highly sensitive and viable alternative to working with radioactive, ^14^C-labeled polymers.

#### Soil Incubations

Incubation of the unreacted PEGs in the *t*_0_ solution resulted in only very low mineralization rates with a maximum of approximately 0.035% ^13^C h^–1^ after 20 h, after which mineralization rates decreased to very low and approximately constant values over the rest of the incubation (Figure 3c). Raw δ^13^C values in CO_2_ of this treatment remained consistently above those of the soil blanks, indicating that the low mineralization rates were reliably measured (Section S6, Supporting Information, for discussion of measurement signal-to-noise). The final ^13^C_Mineralized_ was only 1.25% after 150 days of incubation (Figure 3d), implying that the initial PEG molecules with MWs around 6400 Da did not readily biodegrade in the tested soil. Conversely, soil incubations of PEGs from the *t*_1_, *t*_2_, and *t*_3_ solutions showed increasing mineralization rates and higher rate maxima at approximately 20 h of incubation with increasing ^•^OH reaction time. After approximately 4 days of incubation, the ^•^OH-reacted PEGs showed a second, smaller maximum in mineralization rates. At this point, ^13^C_Mineralized_ values had reached 4, 6, and 8% for the *t*_1_, *t*_2_, and *t*_3_ samples, respectively, all of which were much higher than for the *t*_0_ sample (<0.1%) (Figure 3d). The mineralization rate subsequently decreased for all reacted PEGs to low values. We note that distinct increases in the mineralization extents of reacted PEGs at around 40 days of incubation resulted from wetting of the soil to readjust its water content (Figure 3d; see discussion below). Final mineralization extents after 150 days of incubation increased with increasing PEG-^•^OH reaction extents from ^13^C_Mineralized_ = 20 ± 0.8, to 34 ± 3.2, and 45 ± 0.3% for the *t*_1_, *t*_2_, and *t*_3_ samples, respectively (Figure 3d). Since ^13^CO_2_ continued to be formed when incubations were terminated, higher final extents would have been obtained if the soil incubations had been prolonged. As expected, ^13^C_Non‑Mineralized_ in the soils decreased from *t*_0_ to *t*_3_ (Figure 3f). As for the sediment incubations, the mass balances on total PEG-added ^13^C for all soil incubations were closed (i.e., ^13^C_Mineralized_ + ^13^C_Non‑Mineralized_ ranged from 97 ± 2% to 104 ± 4% of PEG-^13^C initially added; Figure 3f).

#### Comparison of PEG Biodegradation in Sediment and Soil

Increasing rates and final extents of PEG mineralization to ^13^CO_2_ with increasing ^13^C-PEG-^•^OH reaction time confirm the hypothesis of increased biodegradability of lower-MW PEGs formed by chain scissions as compared to the initial higher-MW PEGs. The increase in PEG biodegradation upon reaction with ^•^OH was much less pronounced in the sediment (in which even the unreacted PEGs showed high final ^13^C_Mineralized_) than in the soil (in which the unreacted PEGs did not biodegrade). These findings indicate that PEG biodegradation exhibits a much stronger MW dependence over the tested MW range in soils than sediments. Interestingly, the final ^13^C_Mineralized_ obtained in soils closely match the fractions of PEGs with MW < 2000 Da of 21, 35, and 55 wt % for the *t*_1_, *t*_2_, and *t*_3_ samples, respectively, as determined by HPLC-CAD. This finding suggests an upper limit for PEG biodegradation in soils under the given conditions around or slightly above MW = 2000 Da.

The nature of ^13^C_Non‑Mineralized_ in both sediment and soil incubations was not identified but may have included both residual PEG molecules with comparatively high-MW and PEG-derived ^13^C incorporated into microbial biomass. The latter seems plausible for sediment incubations given that even the nonreacted PEGs underwent extensive mineralization. In this case, the slow and continuous ^13^CO_2_ formation in the sediment during the later stages of the incubation likely reflected slow turnover of microbial biomass containing PEG-derived ^13^C. While biomass incorporation of PEG-^13^C may have also occurred during soil biodegradation, the final ^13^C_Non‑Mineralized_ in the soil incubations were too high to be explained only by biomass incorporation: an upper limit for biomass incorporation of around 50% of the total metabolized substrate carbon has been reported for soil microorganisms^44^ (i.e., maximum carbon use efficiencies around 0.5). It is therefore likely that a significant fraction of ^13^C_Non‑Mineralized_ in soil was present in PEG molecules with MWs too high to be microbially accessible.

Slower and lower final PEG biodegradation in the soil than the sediment may reflect a lower abundance and/or activity of microorganisms biodegrading high-MW PEGs in the soil than sediment as well as higher constraints on the bioavailability of high-MW PEGs in the soil than in the sediment. The first explanation is supported by previous work showing that low-MW PEGs can be utilized as substrates by a broad range of microorganisms, while PEGs with MWs above 4000 Da typically require specific microorganisms or even symbiotic mixed cultures.^12,45,46^ It is conceivable that the latter were less abundant in the soil. Differences in the cell wall architecture and membrane rigidities between soil and sediment microorganisms may also have contributed to the more stringent MW-cutoff for microbial uptake in soils.^47,48^ The second explanation of constrained PEG bioavailability in soils may have resulted from spatial separation between the high-MW PEGs and the specific biodegraders as well as only slow cellular uptake of the higher-MW PEGs by biodegraders.^45^ Higher constraints on bioavailability in soils are consistent with the static (i.e., nonstirred) nature of the incubations run under unsaturated conditions, likely resulting in stronger adsorption and slower diffusional transport of higher-MW PEGs as compared to water-saturated and continuously stirred sediment incubations that likely allowed for high transfer rates of PEG molecules to cell surfaces. Constrained bioavailability of high-MW PEGs in the studied soil is supported by the pronounced increase in mineralization rates that resulted from soil-rewetting at around 40 days of incubation (Figure 3d), indicating that water-addition facilitated desorption of PEG molecules from particle surface and diffusion to microbial cells. These results highlight the need for future studies to elucidate microbial uptake pathways of PEGs (and other WSPs) and intracellular enzymatic processing.^21,49^

## Environmental Implications

This study reveals that PEG reaction with photochemically produced ^•^OH in sunlit environments (lakes, rivers, and agricultural ditches, possibly including also exposed leaf and mineral surfaces) shifts the MW distribution of PEGs to smaller MW molecules with enhanced biodegradability. Considering PEG reaction with ^•^OH in sunlit environments is therefore important for accurately assessing PEG fate and environmental half-lives. While low-MW PEGs were readily taken up and metabolized by microorganisms in soil and sediment, rendering them biodegradable, higher MW PEGs with otherwise identical chemistry remained rather stable in soils. The latter may reflect both limited bioavailability of high-MW PEGs as well as lower abundance and/or activity of special microorganisms capable of biodegrading high-MW PEGs.

Beyond PEGs, this work highlights the importance of considering WSP chain scission reactions and the resulting formation of lower-MW products in advancing a robust understanding of WSP stability, particularly when focusing on biodegradation as the ultimate transformation end point. The probabilistic Monte Carlo simulation model developed and validated herein provides a versatile tool to predict the effect of chain scissions in WSPs on MW distributions. This model can readily be modified to be applicable to WSPs undergoing position-specific chain scission reactions, such as those driven by abiotic or enzymatic hydrolysis of specific bonds in the polymer backbone. In addition, the HPLC-CAD-HRMS methodology presented in this work opens possibilities of how MW-distributions of PEGs are analytically accessible in WSP fate studies. Future work on the environmental fate of WSPs should systematically investigate the MW-dependence of biodegradation of major WSP classes across terrestrial and aquatic environments and, if differences exist between WSPs and receiving environments, elucidate the underlying mechanisms.

## Supplementary Material



## Notes

Detailed reaction rate modeling for hydroxyl radical attack on PEG (Section S1); description of analytical methods and selected chromatograms from HPLC-CAD and HR-MS analyses (Section S2); properties of soil used in incubation experiments (Section S3); schematic and description of the automated incubation system coupled to CRDS analysis for ^13^CO_2_ measurements (Section S4); EA-IRMS-based quantification of residual ^13^C in soil and sediment (Section S5); signal-to-noise analysis of low-level ^13^CO_2_ signals (Section S6); a total of six Figures S1–S6 (PDF)

## ⊥

Department of Biotechnology, Delft University of Technology, 2628 Delft, The Netherlands

## #

Department of Environmental Analytical Chemistry, Helmholtz-Centre for Environmental Research (UFZ), 04318 Leipzig, Germany.
