# Supplementary material for: Photochemical Chain Scissions Enhance Polyethylene Glycol Biodegradability: from Probabilistic Modeling to Experimental Demonstration
Source: Environ Sci Technol. 2025 Aug 15;59(33):17773–84. doi: 10.1021/acs.est.5c03567 (PMC12392441; doi:10.1021/acs.est.5c03567)
Supplement: Supplementary file 1 [file es5c03567_si_001.pdf]

Supplementary Information

Photochemical Chain Scissions Enhance Polyethylene Glycol Biodegradability: From Probabilistic Modelling to Experimental Demonstration

Kevin Kleemann[a], Madalina Jaggi[b], Stefano M. Bernasconi[b], Robert Alexander Schmitz[a,c], Andreas Künkel[d], Carsten Simon[a,e,f], Kristopher McNeill[a], Glauco Battagliarin[d], and Michael Sander[a*]

[a]Institute of Biogeochemistry and Pollutant Dynamics, ETH Zurich, 8092 Zurich, Switzerland

[b]Geological Institute, Department of Earth Sciences, ETH Zurich, 8092 Zurich, Switzerland

[c]*current address*: Department of Biotechnology, Delft University of Technology, 2628 Delft, The Netherlands,

[d]BASF SE, Materials and Formulation Research, Carl-Bosch-Strasse 38, 67056 Ludwigshafen, Germany

[e]Eawag, Swiss Federal Institute of Aquatic Science & Technology, 8600 Duebendorf, Switzerland

[f]*current address*: Department of Environmental Analytical Chemistry, Helmholtz-Centre for Environmental Research (UFZ), 04318 Leipzig, Germany

*Corresponding author:
Michael Sander
Email: michael.sander@env.ethz.ch
Phone: 0041-44 632 8314

Summary: 13 pages, 6 figures, 0 tables

Section S1. Molecular weight-dependence of the rate constant for PEG reaction with •OH in Monte Carlo simulations. Reaction rate constants between PEG molecules and •OH are dependent on the chain length of the PEG molecules (i.e., number of repeat (monomeric) units n, which we subsequently refer to as degree of polymerization). This reaction has two distinct kinetic regimes:[1] the reaction-limited regime for small PEG molecules ($n \leq 30$) and the diffusion-limited regime for large PEG molecules ($n > 30$). The transition between the reaction-limited and the diffusion-limited regimes has previously been shown to occur around $n = 30$.[1]

In the reaction-limited regime, the reaction rate constant k_P (M^{-1} s^{-1}) of a PEG molecule is directly proportional to its number of monomeric units n (**Equation S1**):

$$\boldsymbol{k_P(n) = 0.8 \cdot k_M \cdot n, \qquad \text{for } 1 \leq n \leq 30} \qquad \textbf{Eq. S1}$$

where k_P (M^{-1} s^{-1}) is the rate constant for PEG reacting with •OH, and k_M (M^{-1} s^{-1}) is the reaction rate constant for a monomeric unit. The proportionality constant of 0.8 was selected for PEG based on its use in a previous publication on the reaction between benzophenone radicals and n-alkanes of various lengths.[2]

With increasing n at $n > 30$, the reaction becomes increasingly diffusion-limited due to the increasing number of reaction sites per PEG molecule (as reflected in an increase in the effective rate constant k_P). At the same time, the larger PEG molecules undergo coiling in solution. This coiling lowers the rate constant of the reaction between •OH and a monomeric unit of the PEG chain, k_M, because the reaction is preferably occurring on the surface of the PEG coil. This effect can be accounted for by incorporating the effective reaction radius of the

PEG molecule into the reaction rate constant using the Smoluchowski equation for diffusion-limited reactions in dilute solutions (**Equation S2**):[1]

$$\boldsymbol{k_{\mathrm{P}}(n) = 4\pi(R_{\cdot\mathrm{OH}} + R_{\mathrm{PEG}}) \cdot (D_{\cdot\mathrm{OH}} + D_{\mathrm{PEG}}) \cdot N_A = 4\pi(R_{\cdot\mathrm{OH}} + R_{\mathrm{PEG}}) \cdot \left(\frac{k_{\mathrm{B}}T}{6\pi\eta R_{\cdot\mathrm{OH}}} + \frac{k_{\mathrm{B}}T}{6\pi\eta R_{\mathrm{PEG}}}\right) \cdot N_A,\ \ \text{for } n > 30} \qquad \textbf{Eq. S2}$$

where $R_{\cdot\mathrm{OH}}$ and R_{PEG} (dm) are the hydrodynamic radii of •OH and the coiled PEG molecule, respectively, T (K) is the absolute temperature, η (kg dm^{-1} s^{-1}) is the dynamic viscosity of water, N_A (mol^{-1}) is the Avogadro constant, and $D_{\cdot\mathrm{OH}}$ and D_{PEG} (dm^2 s^{-1}) are the effective diffusion coefficients of the •OH and the PEG molecule, respectively. Since $R_{\cdot\mathrm{OH}} << R_{\mathrm{PEG}}$ and $D_{\cdot\mathrm{OH}} >> D_{\mathrm{PEG}}$, **Equation S2** simplifies to **Equation S3**:

$$\boldsymbol{k_{\mathrm{p}}(n) = 4\pi \cdot R_{\mathrm{PEG}} \cdot D_{\cdot\mathrm{OH}} \cdot N_A,\ \ \text{for } n > 30} \qquad \textbf{Eq. S3}$$

The hydrodynamic radius of the coiled PEG chain, $\boldsymbol{R_{\mathrm{PEG}}}$, scales in the Flory theory for good solvents as $\boldsymbol{R_{\mathrm{PEG}}} \propto n^{0.6}$.[1] Experimental observations found $\boldsymbol{k_{\mathrm{p}}}(n) \propto n^{0.56}$ for PEGs with n > 30, which is subsequently used as a constant parameter for the n-dependent rate constants in the diffusion-limited regime.[1] **Figure S1** presents the relationship between the degree of polymerization (n) and two •OH reaction rate constants: the polymer reaction rate constant k_{P} and the monomer reaction rate constant k_{M}. For PEG molecules with a few repeating units (i.e., $n < 30$), k_{P} increases linearly.

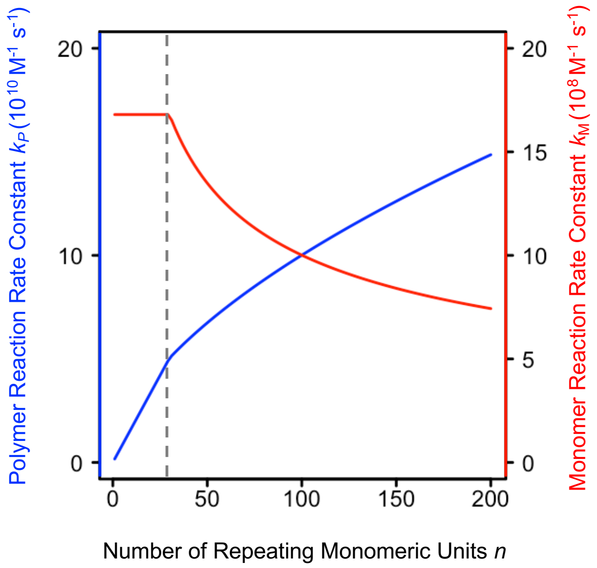

Figure S1. Reaction rate constants of polyethylene glycol (PEG) with hydroxyl radical (•OH) as a function of the degree of PEG polymerization (n). The blue curve represents the polymer reaction rate constant k_P (M^{-1} s^{-1}) and the red curve represents the monomer reaction rate constant, k_M (M^{-1} s^{-1}). The vertical dashed line corresponds to the transition from the reaction-limited regime ($n < 30$) to a diffusion-limited regime ($n > 30$).

Section S2. High-pressure liquid chromatography (HPLC) coupled to parallel charged aerosol detection (CAD) and high-resolution mass spectrometry (HR-MS). Separation of PEG molecules and PEG transformation products was performed using the Vanquish™ Core HPLC System (Thermo Scientific) equipped with a HALO C18 column (90 Å, 2.7 µm, 4.6 × 100 mm) and coupled to a Vanquish™ Charged Aerosol Detector (CAD). The column temperature was set to 50°C. The mobile phase consisted of water (Phase B) and acetonitrile (Phase A), with a flow rate of 1 mL/min. The gradient program was as follows (with the missing percentage to 100% being phase A): 78% B for 120 min, 73% B for 180 min, 69% B for 300 min, 67% B for 200 min, 30% B for 20 min, and finally 73% B for 5 min. The injection volume was 100 µL. For the CAD, the evaporation temperature was set to 50°C.

The complete chromatograms of the four experimental PEG solution samples (i.e., unreacted t_0 sample containing the initial PEG and the incrementally reacted solutions at t_1, t_2, and t_3, containing transformed PEG) are shown in **Figure S2**.

In addition to detection by CAD, the column effluent was also passed to a parallelly operated QExactive HF Orbitrap™ HR-MS system (Thermo Scientific, Switzerland), equipped with an electrospray ionization (ESI) source. The system was calibrated using Pierce™ ESI calibration solutions (Thermo Scientific). In the heated electrospray ionization (HESI) source, a spray voltage of 2.7 kV was applied, with a capillary temperature of 320°C, and the auxiliary gas heater was set to 400°C. All analyses were conducted in positive ionization mode. The HR-MS spectra of PEG_{32}, PEG_{41}, and PEG_{61} from samples t_0 and t_3 are shown in **Figure S3**.

Carbon-13 Nuclear Magnetic Resonance Spectroscopy (^{13}C NMR). We analyzed the four experimental solutions t_0 to t_3 containing unreacted and reacted ^{13}C-PEG by ^{13}C-NMR (Bruker Avance III 400 MHz NMR). ^{13}C NMR spectra were acquired on a Bruker Avance III 500 MHz spectrometer in D_2O using 1,024 scans (NS), a relaxation delay (D1) of 2 s, a 10 µs 90° pulse (P1), a spectral width of 238.9 ppm, an acquisition time of 1.36 s, 4 dummy scans (DS), and a line broadening (LB) of 1 Hz. The corresponding NMR spectra are shown in **Figure S4**.

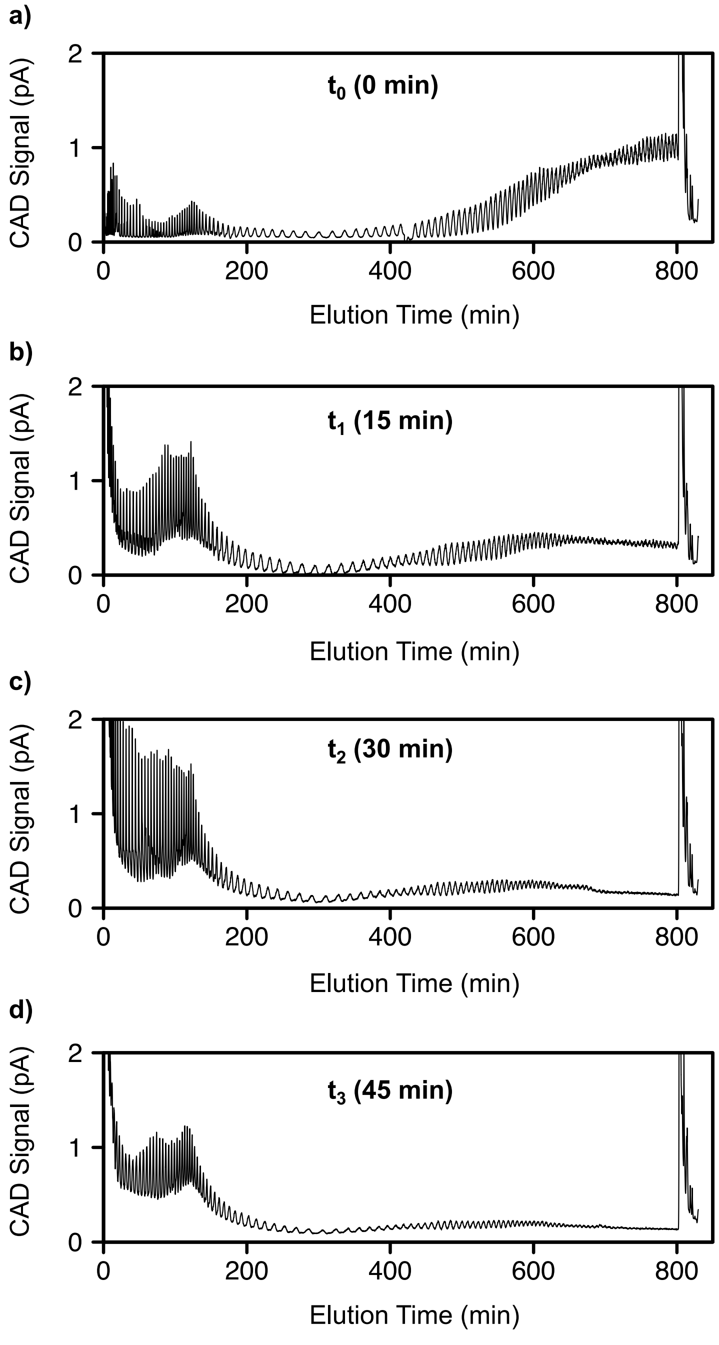

Figure S2. Chromatograms from the high-pressure liquid chromatography analysis coupled to a charged aerosol detector (HPLC-CAD) of ^{13}C-polyethylene glycol (PEG) solutions after reaction times of PEG with hydroxyl radical (•OH) for **a)** t_0= 0 min (unreacted solution), **b)** t_1= 15 min, **c)** t_2= 30 min, and **d)** t_3= 45 min.

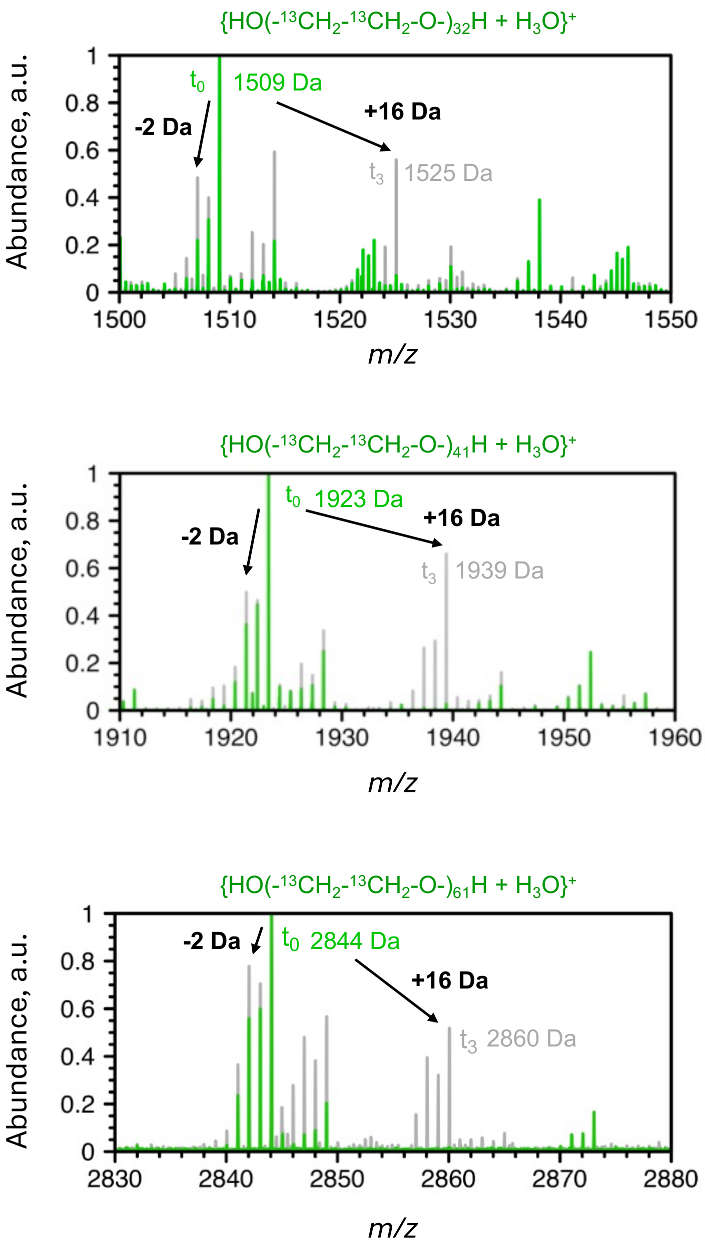

Figure S3. Mass spectra (ion abundance over mass-to-charge ratio, m/z) of selected ^{13}C-polyethylene glycol (PEG) molecules after reaction times of $t_0 = 0$ min (green; unreacted initial solution) and $t_3 = 45$ minutes (grey) between PEG and hydroxyl radical (•OH). The black arrows specify the increases and decreases in masses.

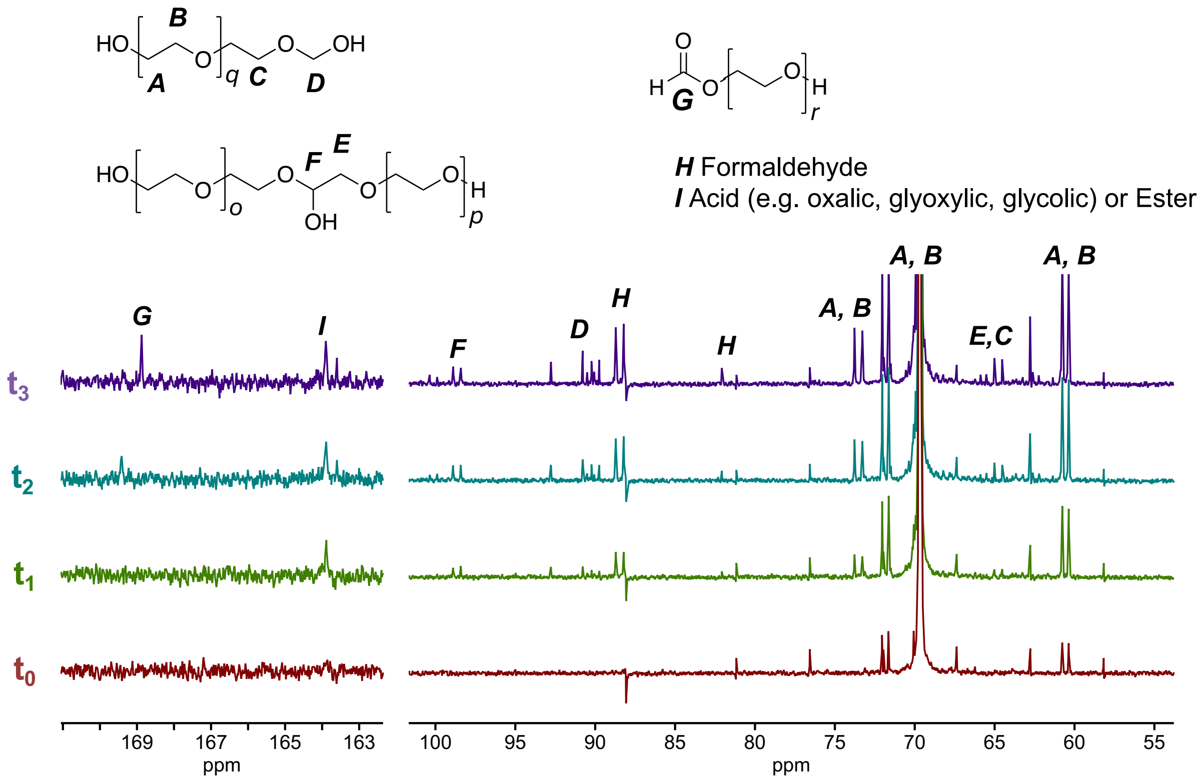

Figure S4. ^{13}C nuclear magnetic resonance (NMR) spectra of ^{13}C-polyethylene glycol (PEG) and their reaction products after $t_0 = 0$ min (red; unreacted solution), $t_1 = 15$ min (green), $t_2 = 30$ min (cyan), and $t_3 = 45$ min (purple) of reaction with hydroxyl radical (•OH). Peaks of structural groups **A-I** and corresponding chemical structures are specified above.

Section S3. Properties of soil LUFA 6S

Soil LUFA 6S used in the incubations had an organic carbon content of 1.55 wt%, a nitrogen content of 0.18 wt%, a pH of 7.3, and a cation exchange capacity of 18.7 meg/100 g, as specified by the Landwirtschaftliche Untersuchungs- und Forschungsanstalt Speyer, Germany. The soil is classified as a clay soil.

Section S4. Incubation setup for automated mineralization measurements

We followed mineralization of ^{13}C-labeled polyethylene glycol (PEG) from the different experimental solutions (i.e., samples t_0 to t_3) using a previously described incubation system[3] with slight modifications. A schematic diagram of the setup of the modified system is provided in **Figure S5**. Ambient air from the laboratory pressurized air system was continuously pushed into a large Schott bottle (2 L) to dampen small fluctuations in the CO_2 concentrations in the air system before being pumped through the incubation system. Following the larger Schott

bottle, the air was passed through two closed glass bottles (1.5 L each) containing MilliQ water. These bottles served to humidify the air stream by positioning the gas influx below the water surface, resulting in air bubbling through the water, and the efflux at the top of the bottles. This humidification step served to minimize evaporative water loss in the subsequent soil and sediment incubation bottles. The humidified air was led through an additional large Schott bottle (2 L) with a small opening for overpressure release. Afterwards, the air was split into a total of 30 separate gas lines, each of which was connected to a 100 mL Schott bottle containing either soil or sediment for incubation (i.e., two pairs of 15 bottles for each soil and sediment; triplicate incubation bottles for only background (i.e., soil or sediment without PEG added) and triplicate incubation bottles with added t_0, t_1, t_2, and t_3 PEG solutions). The incubation bottles were sealed with gas-tight lids, equipped with inlets, outlets, and septa. The latter allowed for water and sample addition without having to open the incubation bottles.

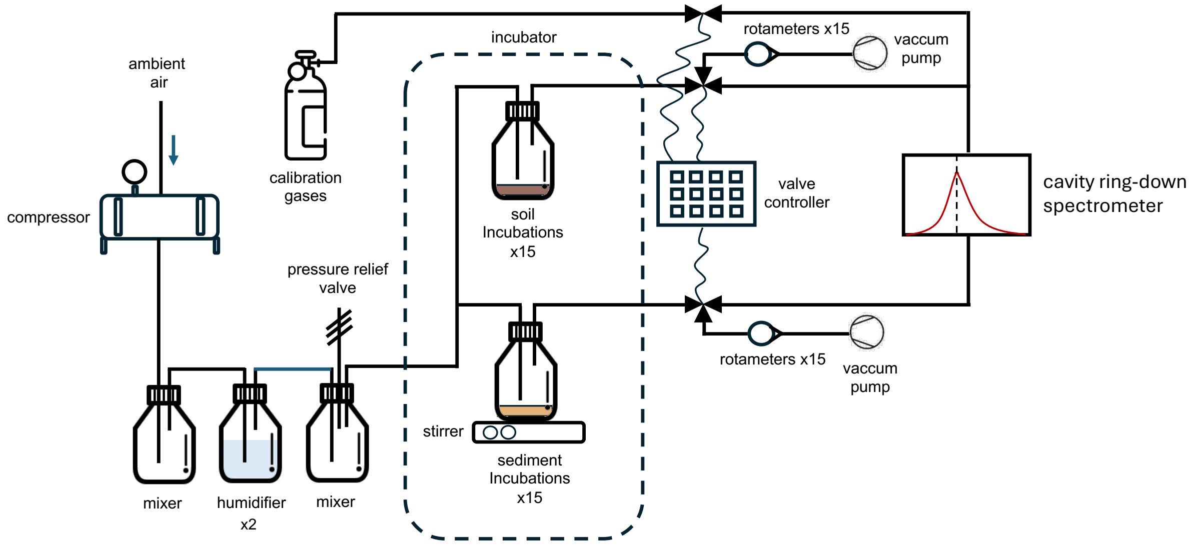

Figure S5. Schematic of the automated soil and sediment incubation system coupled to the on-line analysis of concentrations of $^{13}CO_2$ and $^{12}CO_2$ in the bottle gas efflux using cavity ring-down spectroscopy.

The incubation bottles were housed in a laboratory incubator (Binder BD400), maintaining a constant temperature of 20±1 °C. The outlets of the bottles were connected to a set of thirty solenoid valves which were controlled by an automated and programmable logic controller (custom made). At any given time, one valve was actuated to direct the efflux gas from the selected incubation bottle to a cavity ring-down spectroscopy analyzer (CRDS, model G2201i, Picarro) for real-time measurement of $^{13}CO_2$ and $^{12}CO_2$ concentrations at a volumetric flow rate of 22.4 mL min^{-1}. The efflux gas from the remaining 29 non-selected incubation bottles was continuously pulled through individual flow rotameters towards an external diaphragm pump and into exhaust. The volumetric flow during this flushing was also set to approximately 22.4 mL min^{-1} by use of the rotameters to ensure that the CO_2 concentration in the headspace of each incubation bottle was in steady state with the flow rate at the time that the incubation bottle was analyzed by the CRDS. Periodically, the CRDS on the system was calibrated using standard gases with known concentrations and isotopic composition of CO_2. The mineralization rates and extents from the measured $^{13}CO_2$ and $^{12}CO_2$ concentrations in the efflux gas were calculated as previously described.[3] We used ^{13}C atom% to calculate the fractional contributions of carbon from the added ^{13}C-labelled PEG. We assumed a total carbon content of 56.48 wt% for the ^{13}C-PEG.

Section S5. Quantification of residual, PEG-added ^{13}C in soil and sediment at the end of the incubation.

At the end of the incubation, all incubation bottles were frozen and subsequently freeze-dried for 48 hours. The freeze-dried soil and sediment were removed from each bottle and milled (MM400 oscillatory ball mill from Retsch GmbH & Co, frequency of 30 Hz for 1 minute) for sample homogenization. Subsequently, a mass of approximately 4 mg of each milled soil or sediment was weighed directly into tin capsules for elemental analysis.

The carbon isotopic composition ($\delta^{13}C$ relative to VPDB) and carbon content (%C by mass) of each soil or sediment sample were analyzed using an elemental analyzer (EA) (Thermo Fisher FlashEA 1112) connected to a continuous flow interface (Thermo Fisher ConFlo IV) and an isotope-ratio mass spectrometer (IRMS) (Thermo Fisher Delta V Plus). Data collection was performed using Isodat (version 3.0), and helium was used as the carrier gas at a flow rate of 80 mL min^{-1}. For analysis, the tin capsules were placed in a MAS 200 autosampler and introduced sequentially to the oxidative column of the EA (with chromium oxide and silvered cobaltous oxide at 1020°C). After introducing the sample, oxygen was injected for combustion at a flow rate of 175 mL min^{-1} for 3 seconds. The resulting gases passed through a reductive column (packed with elemental copper, at 650°C), followed by drying with magnesium perchlorate. A GC column (2 m long, packed with Porapak QS 50/80 mesh, at 45°C) was used to separate CO_2 from other gases before introducing it into the IRMS. Samples were compared with a CO_2 reference gas with a known $\delta^{13}C$-CO_2 of −28.23‰.

To ensure linearity in the EA-IRMS analysis, organic compounds with known, varying isotopic compositions were also analyzed, including nicotinamide ($\delta^{13}C$ = −31.2‰), peptone ($\delta^{13}C$ = −15.6‰), and a custom glucose mixture ($\delta^{13}C$ = 100‰). The glucose standard was prepared by mixing non-labeled glucose with $^{13}C_6$-glucose, and the final isotopic value ($\delta^{13}C$ = 100 ± 1.2‰) was confirmed by replicate EA-IRMS analyses. We quantified the non-mineralized PEG-added ^{13}C that remained in the soil or sediment (i.e., $^{13}C_{Non\text{-}Mineralized}$) samples using EA-IRMS data and the mathematical procedure previously described.[3]

Section S6. Signal-to-noise analysis for incubation of non-treated PEG (t_0) in Soil 6S

Signal-to-noise (S/N) ratios were calculated by dividing the daily-averaged, background-corrected sample signal (i.e., the difference between the daily average $\delta^{13}C$ value of the sample and the daily average $\delta^{13}C$ value of the blanks) by the standard deviation of blank replicates measured on the same day. This analysis was performed exemplarily for the incubation with

the lowest signal — non-treated PEG (t_0) incubated in Soil 6S — to assess whether the observed $^{13}CO_2$ signals were reliably above the background and could be detected and quantified. The distribution of blank variability showed that most daily standard deviations ranged between 0.03‰ and 0.07‰ (median: 0.047‰), with a few elevated values up to 2.72‰. The resulting daily S/N ratios are shown in Figure S6. The sample signal remained well above background noise throughout the incubation period, with S/N ratios typically between 20 and 50.

Accurate quantification even of the t_0 treatment in soil is further demonstrated by the closed mass balance on PEG-added ^{13}C also in this incubation (see Figure 3f in manuscript).

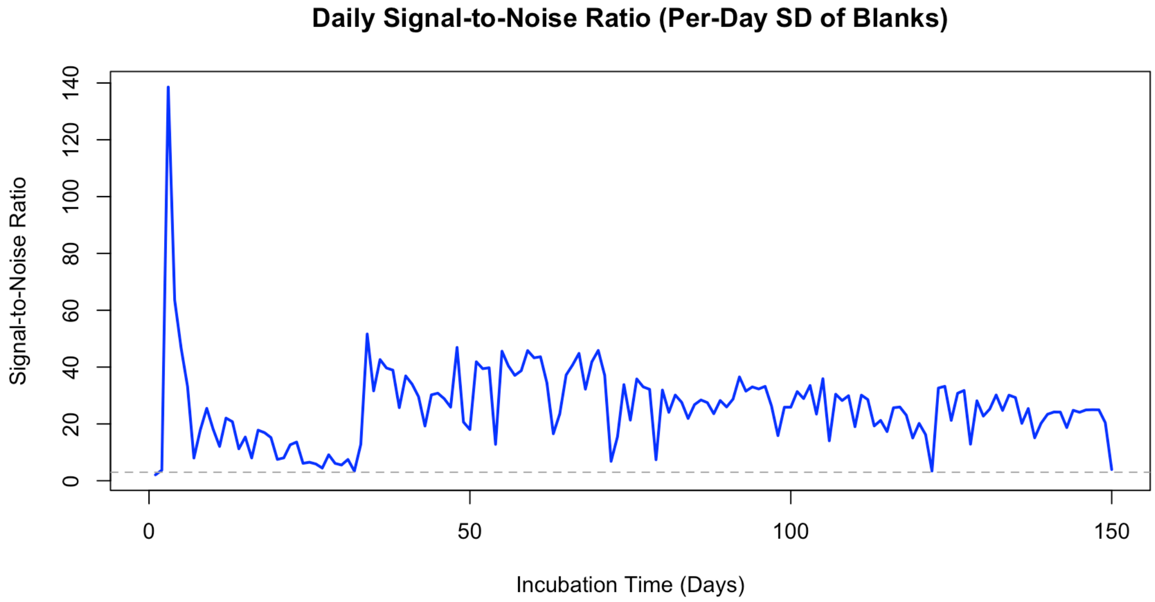

Figure S6. Daily signal-to-noise (S/N) ratios for the incubation of non-treated PEG (i.e., t_0) in Soil 6S. S/N ratios were calculated as the daily-averaged, background-corrected sample signals — that is, the difference between the daily average $\delta^{13}C$ values of the sample CO_2 and the daily average $\delta^{13}C$ value of the blanks CO_2 — divided by the standard deviation of concurrently measured blank replicates. The dashed horizontal line at S/N = 3 indicates a conservative detection threshold. This analysis was performed exemplarily for the lowest-signal sample to confirm that measured signals in all systems remained well above background variability throughout the incubation period.

References

(1) Matheson, M. S.; Mamou, A.; Silverman, J.; Rabani, J. Reaction of Hydroxyl Radicals with Polyethylene Oxide in Aqueous Solution. *J. Phys. Chem.* **1973**, *77* (20), 2420–2424. https://doi.org/10.1021/j100639a011.

(2) Winnik, M. A.; Maharaj, U. Photoreaction of Benzophenone with the N-Alkanes: A Model for Biomolecular Reactions of Polymers. *Macromolecules* **1979**, *12* (5), 902–905. https://doi.org/10.1021/ma60071a022.

(3) Nelson, T. F.; Baumgartner, R.; Jaggi, M.; Bernasconi, S. M.; Battagliarin, G.; Sinkel, C.; Künkel, A.; Kohler, H.-P. E.; McNeill, K.; Sander, M. Biodegradation of Poly(Butylene Succinate) in Soil Laboratory Incubations Assessed by Stable Carbon Isotope Labelling. *Nat Commun* **2022**, *13* (1), 5691. https://doi.org/10.1038/s41467-022-33064-8.
